# Evaluation of the Influence of Biological Factors during the Course of Treatment in Patients with Ovarian Cancer

**DOI:** 10.3390/ijerph191710516

**Published:** 2022-08-24

**Authors:** Daria Schneider-Matyka, Edyta Skwirczyńska, Maria Gaur, Beata Hukowska-Szematowicz, Sebastian Kwiatkowski, Marzena Mikla, Elżbieta Grochans, Aneta Cymbaluk-Płoska

**Affiliations:** 1 Department of Nursing, Pomeranian Medical University in Szczecin, 71-210 Szczecin, Poland; 2 Department of History of Medicine and Medical Ethics, Pomeranian Medical University in Szczecin, 70-205 Szczecin, Poland; 3 Department of Gynecological Surgery and Gynecological Oncology of Adults and Adolescents, Pomeranian Medical University in Szczecin, 70-111 Szczecin, Poland; 4 Institute of Biology, University of Szczecin, 71-412 Szczecin, Poland; 5 Molecular Biology and Biotechnology Center, University of Szczecin, 71-412 Szczecin, Poland; 6 Department of Obstetrics and Gynecology, Pomeranian Medical University in Szczecin, 70-111 Szczecin, Poland; 7 Department of Nursing, University of Murcia, Campus de Ciencias, El Palmar, 30120 Murcia, Spain; 8 Group ENFERAVANZA, Murcian Institute of Biosanitary Research (IMIB), 30120 Murcia, Spain

**Keywords:** ovarian cancer, β-endorphin, serotonin, serum, the overall survival, disease-free time

## Abstract

The aim of this study was to evaluate the influence of β-endorphins and serotonin on the course of treatment, disease-free time, and overall survival of patients with ovarian cancer. This study may contribute to the identification of modifiable factors that may influence the treatment of ovarian cancer. The research was carried out in a group of 162 patients of which 139 respondents were included in the research; ovarian cancer was diagnosed in 78 of these patients. The study consisted of three stages. In the first stage of diagnostics, a survey among the patients was carried out. In the second stage—5 mL of blood was collected from each patient (*n* = 139) in the preoperative period to determine the concentration of β-endorphin and serotonin. In the third stage—blood samples were collected from those patients who had completed chemotherapy treatment or had surgery. Concentrations of β-endorphin and serotonin were measured by the Luminex method, using the commercial Luminex Human Discovery Assay kit. The average age of the patients was 62.99 years. The level of β-endorphin significantly differs among patients diagnosed with ovarian cancer and among patients in the control group (202.86; SD—15.78 vs. 302.00; SD—24.49). A lower level of β-endorphins was found in the patients with a recurrence of the neoplastic process compared to those without recurrence (178.84; SD—12.98 vs. 205.66; SD—13.37). On the other hand, the level of serotonin before chemotherapy was higher in the group of people with disease recurrence compared to those without recurrence (141.53; SD—15.33 vs. 134.99; SD—10.08). Statistically significantly positive correlations were found between the level of β-endorphin and both disease-free time (β-endorphin levels before chemotherapy: rho Spearman 0.379, *p* < 0.027; β-endorphin levels after chemotherapy: rho Spearman 0.734 *p* < 0.001) and survival time (β-endorphin levels before chemotherapy: rho Spearman 0.267, *p* < 0.018; β-endorphin levels after chemotherapy: rho Spearman 0.654 *p* < 0.001). 1. The levels of serotonin and β-endorphin levels are significantly related to ovarian cancer and change during treatment. 2. High mean preoperative concentrations of β-endorphins were significantly related to overall survival and disease-free time.

## 1. Introduction

Worldwide, ovarian cancer is the seventh most common cancer in women and the eighth most common cancer-related cause of death, with a five-year survival rate of less than 45%. While age-standardized case rates are stable or falling in most high-income countries, they are rising in many low and middle-income countries. Moreover, as life expectancy increases, the number of cases diagnosed each year increases. To control ovarian cancer we need to understand the causes. This will enable better identification of those most at risk for whom screening tests may be targeted, and potentially modifiable causes to provide the opportunity to intervene and reduce disease rates [1].

Ovarian cancer is a serious epidemiological problem. Its symptoms are unusual and can be easily overlooked [2]. Ovarian cancer is characterized by a propensity for local spreading and increased production of peritoneal fluid. In over 70% of cases, patients are diagnosed after cancerous lesion on the pelvis and/or the entire abdominal cavity have spread [2].

Surgery is a basic element of the therapeutic management of patients with ovarian cancer. The goal of surgical treatment is to obtain optimal cytoreduction, which significantly extends the survival time. Sometimes it is impossible to remove all of the widespread abdominal lesions, especially where leaving a residual disease is a poor prognostic factor in patients with ovarian cancer. Surgical interventions are complemented by adjuvant chemotherapy. By default, six cycles of paclitaxel and platinum are delivered at three-week intervals. The standard treatment regimen achieves remission in approximately 80% of cases, but despite the initial effectiveness, most patients experience a relapse within the first 5 years.

Ovarian cancer is called the silent killer because it can progress quickly and be asymptomatic at the beginning of the disease. A patient with neoplastic disease, and in particular with such an insidious disease, is exposed to emotional states that, under unfavorable conditions, may lead to increased anxiety as well as depression and all the consequences of these conditions. Chronic stress has been reported to induce a number of biological responses involving the nervous, endoc,rine and immune systems that are associated with the process of carcinogenesis. There is an increased synthesis of substances such as catecholamines, glucocorticosteroids as well as growth hormones, inflammatory cytokines, and endogenous opioids.

Endorphins are natural morphine-like substances, mainly β-endorphins. They abundantly bind to μ receptors present on immune cells, i.e., macrophages, T and B lymphocytes, monocytes, and natural killers (NK) cells, which cause the production of anti-inflammatory cytokines: IL-18, IL-10, interferon-γ (IFN-γ). NK cells constitute the natural first line of antitumor and antiviral defense through the production of IFN-γ, opsonin, and granzyme-B [3,4,5,6]. β-endorphins induce analgesia through the action of an inhibitory substance P, a neurotransmitter of pain in the peripheral nerves, by presynaptic binding to the μ receptor. β-endorphins induce euphoria, satisfaction, and an analgesic effect by inhibiting the gamma-aminobutyric acid (GABA) neurotransmitter and stimulating the release of dopamine involved in analgesic activity, euphoria, and stress killer activity [7,8,9,10,11,12]. Additionally, β-endorphins inhibit chronic mental stress by inhibiting the hypothalamic-pituitary-adrenal (HPA) axis by the autonomic nervous system (ANS). β-endorphins lower stress by inhibiting the secretion of neurohormones such as cortisol, adrenocorticotropic hormone (ACTH), and norepinephrine. These neurohormones activate inflammatory mediators such as IL-1, TNF-α, and IL-6, which additionally activate nuclear factor kappa B (NF-ĸB) and signal transducer and activator of transcription 3 (STAT-3)—transcription factors involved in tumor progression [13,14].

Serotonin or 5-hydroxytryptamine (5-HT) is a neurotransmitter known to affect emotions, behavior, and cognition. It is known that the penetration between the immune cells and the nervous system via serotonin and its receptors (5-HTR) in the tumor microenvironment and lymphatic secondary organs influence the pathogenesis of cancer. However, the molecular mechanism—changes in phenotype and function—of congenital and adaptive immune cells by serotonin are not well studied [15]. The cortical, limbic, midbrain, and hindbrain regions receive projections produced by serotonin neurons in the brainstem. Serotonin affects the processes related to mood, perception, anger, memory, attention, and sexuality. Thus, it is noticeable that the vast majority of everyday behavior is more or less regulated by this hormone [16]. Whereas studies on the effect of serotonin indicate its dichotomous role in tumor progression depending on the serotonin serum concentration. It has been shown that too high serotonin levels can lead to the development of aggressive forms of cancer through 5-HT1 and 5-HT2 receptors. On the other hand, studies indicate that low serotonin concentrations may inhibit blood flow to the brain, preventing tumor progression [17].

The aim of this study was to evaluate the influence of β-endorphin and serotonin on the course of treatment, disease-free time, and overall survival of patients with ovarian cancer.

## 2. Materials and Methods

The research was conducted on a group of 162 patients who had been diagnosed and treated from 2020 to 2021 at the Department of Surgical Gynecology and Gynecological Oncology of Adults and Adolescents of the Pomeranian Medical University in Szczecin. The following are the criteria for inclusion in the study:

- Sex
- Age 18–65 years
- Initially, patients were recruited for examinations based on imaging examinations including tomography and ultrasonography. Ultimately, only patients with histopathologically confirmed ovarian cancer participated in the study
- No history of psychiatric disease

The mental health of the respondents was assessed using a screening tool—the Primary Care Evaluation of Mental Disorders Patient Health Questionnaire 9 (PRIME-MD PHQ-9).

Criteria for exclusion from the research are:

- coexistence of other neoplasms
- diagnosed endometriosis
- coexistence of collagen diseases
- psychiatric treatment
- psychological therapy before the diagnosis of changes in the ovary

The research was conducted according to ethical standards and the Declaration of Helsinki. The study protocol was approved by the Bioethics Committee of the Pomeranian Medical University in Szczecin, Poland (KB-0012/97/2020). Informed consent was obtained from the study participants. Each of the study participants was informed about the possibility of withdrawing from the study at any time.

The study was carried out from a group of 162 patients, of which 15 did not meet the inclusion criterion, in three cases it was not possible to secure biological material, and 5 incorrectly filled in the questionnaire, leaving 139 patients enrolled in the study.

On the basis of ultrasonography, computed tomography and the results of histopathological examinations, 78 of the patients had been diagnosed with ovarian cancer and so were included in the study group, and the remaining 61 patients with benign neoplastic lesions and simple ovarian cysts were included in the control group. Of the 78 patients diagnosed with ovarian cancer, 67 were diagnosed with serous ovarian cancer, and 11 with non-serous ovarian cancer. According to FIGO classification, 68 were classified as 3rd and 4th stage cancer and 10 were classified as 1st and 2nd stage. A total of 43 patients were classified as grade 3, 29 patients with grade 2, and 5 patients with grade 1. Recurrence occurred in 33 patients, according to FIGO classification, 32 were classified as 3rd and 4th stage cancer and 1 was classified as 2nd stage [18].

The study consisted of three parts. In the first part, a diagnostic survey among the patients was carried out. A questionnaire was used regarding basic sociodemographic data (age, sex, education, place of residence, marital status, and professional activity) and a standardized questionnaire for assessing psychological factors: Coping Inventory For Stressful Situations (CISS), Stress Sensation Questionnaire (KPS), Beck’s Depression Inventory (BDII), Multidimensional Health Locus of Control A (MHLC A), Multidimensional Health Locus of Control B (MHLC B), General Self-Efficacy Scale(GSES), Satisfaction With Life Scale (SWLS); (Table A1 in Appendix A).

In the second part of the study, 5 mL of blood was collected from each patient (*n* = 139) in the preoperative period to determine the concentration of β-endorphin and serotonin.

In the third part of the study, blood samples were collected from those patients who had completed chemotherapy treatment or had undergone surgery.

Blood for biochemical analysis was collected from a venous vessel (5 mL) according to the procedure for collecting, storing, and transporting biological material from a peripheral vein, between 7.00 and 9.30 a.m. after overnight fasting and a 10-min rest in a sitting position, into Vacutainer tubes (Sarstedt, Nümbrecht, Germany).

Serum concentrations of β-endorphin and serotonin were measured by the Luminex method based on color-coded superparamagnetic spheres coated with analyte-specific antibodies (Luminex Corporation, Austin, TX, USA) using the commercial Luminex Human Discovery Assay (3-plex) kit (R&D Systems, Minneapolis, MN, USA). The treatment was performed according to the manufacturer’s protocol. Briefly, 50 µL of blank standards and samples were added to a 96-well plate and incubated with the microparticle cocktail for 2 h in the dark at room temperature on a horizontal orbital microplate shaker set at 750 rpm. After the incubation time had elapsed, the wells were washed three times with a 1× washing buffer (100 µL/well). In the next step of the procedure, 50 µL of the biotin-antibody cocktail was added to the plate and incubated for 1 h in the dark at room temperature on a horizontal orbital microplate shaker (750 rpm). In the last step of the procedure, streptavidin-PE (50 µL/well) was added to the plate and incubated for 30 min under the same conditions as in the previous steps. Finally, the microspheres on the plate were washed three times, resuspended in a washing buffer (100 µL/well), and read on a Luminex 200 analyzer. Test protein concentrations were calculated from a six-point standard curve.

### Statistical Analysis

In order to verify the hypotheses, statistical analyzes were performed using the IBM SPSS Statistics 25 package. Basic descriptive statistics were analyzed and the Kolmogorov-Smirnov test was used to verify the normality of the distribution of the studied variables, the Spearman’s rank coefficient correlation test to describe the strength of the correlation, the Student’s *t*-test for independent-sample comparisons, and the Student’s *t*-test for paired samples. The classic threshold α = 0.05 was considered the level of significance.

A Kolmogorov-Smirnow test was also performed in order to assess the compliance of the distribution of the analyzed variables with the normal distribution.

The distributions of β-endorphin and serotonin levels in measurement II and the number of months to relapse were similar to the normal distribution. In the case of the other distributions, statistically significant results were recorded, indicating a difference from a normal distribution. In this case, we additionally verified the degree of skewness. When the skewness of the studied distributions was between −2 to +2, it can be assumed that they are not significantly asymmetric compared to the mean which was observed for all the studied quantitative variables, therefore parametric test (the Student’s *t*-test) was used for the statistical analysis [19].

## 3. Results

The average age of the respondents was 62.99 years, 43.39% had higher education, 45.26% had secondary education, and 11.35% had vocational education. A total of 47.3% of the respondents came from a city with more than 100,000 inhabitants, 35.14% came from smaller towns, and the remaining respondents (17.56%) came from rural areas. A total of 69.55% of the respondents remained in a relationship, 30.45% were not married, 31.4% were professionally active, and 68.6% were not professionally active.

The mean concentration of β-endorphin at the first measurement in the patients was 246.37 pg/mL; median 220.90 pg/mL; and standard deviation 53.27. In the second measurement, the mean was 194.31 pg/mL; median 195.89 pg/mL; and standard deviation 18.71 pg/mL. The mean serotonin concentration at the first measurement was 141 pg/mL; median 136.80 pg/mL; and standard deviation 18.15 pg/mL. At the second measurement, the mean serotonin level was 135.24 pg/mL; median 136.70 pg/mL; and standard deviation 20.36 pg/mL. The mean time to relapse among the cancer patients was 16.76 months; the median was 17.5 months; and the standard deviation 5 months. The mean survival time of the subjects with ovarian cancer was 32.64 months; median 36 months; and standard deviation 5.48 months.

The result of the Kolmogorov-Smirnov test is statistically significant, which indicates a lack of compliance with the normal distribution for β-endorphin measurement I, serotonin measurement I, and survival months—Table 1.

β-endorphin concentration was assessed in the study group of patients with diagnosed ovarian cancer (I–II stage according to FIGO) and in the control group of patients with diagnosed benign neoplastic lesions. In the patients with ovarian cancer (I–II stage according to FIGO), the mean concentration of β-endorphin was 223.6 pg/mL, standard deviation of 16.08 pg/mL. Among the patients with a benign neoplastic lesion, the mean concentration of β-endorphin was 302.00 pg/mL, and standard deviation was 24.49 pg/mL. Analysis of the Student’s *t*-test for independent samples showed that the level of β-endorphins significantly differed between the patients diagnosed with ovarian cancer (I–II stage according to FIGO) and the patients from the control group (*p <* 0.003) (Table 2).

β-endorphin concentration was assessed in the study group of patients with diagnosed ovarian cancer (III–IV stage according to FIGO) and in the control group of patients with diagnosed benign neoplastic lesions. In the patients with ovarian cancer (III–IV stage according to FIGO), the mean concentration of β-endorphin was 208.6 pg/mL, and the standard deviation was 14.23 pg/mL. Among the patients with a benign neoplastic lesion, the mean concentration of β-endorphin was 302.00 pg/mL, and the standard deviation was 24.49 pg/mL. Analysis of the Student’s *t*-test for independent samples showed that the level of β-endorphins significantly differed between the patients diagnosed with ovarian cancer (III–IV stage according to FIGO) and the patients from the control group (*p* < 0.001). The strength of the observed effect was very high (Table 3).

β-endorphin concentration was also assessed in the study group of patients with diagnosed ovarian cancer -III–IV stage according to FIGO and in the group of patients with diagnosed ovarian cancer- I–II stage according to FIGO. There was no statistically significant difference in the levels of β-endorphin in the group with ovarian cancer -III–IV stage according to FIGO and in the group with ovarian cancer- I–II stage according to FIGO (*p* < 0342) (Table 4).

The serotonin concentration was assessed in the patients from the study and control groups. In the patients with ovarian cancer (I–II stage according to FIGO), the mean concentration of serotonin was 134.62 pg/mL, standard deviation—10.93 pg/mL. Among the patients with a mild neoplastic lesion, the mean concentration of serotonin was 145.15 pg/mL, standard deviation 22.66 pg/mL. Analysis using the Student’s *t*-test for independent samples showed statistically significant differences between patients with ovarian cancer and the patients with a mild neoplastic lesion (*p* < 0.038)—Table 5.

The serotonin concentration was assessed in the patients from the study and control groups. In the patients with ovarian cancer (III–IV stage according to FIGO), the mean concentration of serotonin was 136.22 pg/mL, standard deviation—11.96 pg/mL. Among the patients with a mild neoplastic lesion, the mean concentration of serotonin was 145.15 pg/mL, standard deviation 22.66 pg/mL. Analysis using the Student’s *t*-test for independent samples showed statistically significant differences between patients with ovarian cancer (III–IV stage according to FIGO) and the patients with a mild neoplastic lesion (*p* < 0.048)—Table 6.

Serotonin concentration was also assessed in the study group of patients with diagnosed ovarian cancer-III–IV stage according to FIGO and in the group of patients with diagnosed ovarian cancer-I–II stage according to FIGO. There was no statistically significant difference in the levels of serotonin in the group with ovarian cancer -III–IV stage according to FIGO and in the group with ovarian cancer-I–II stage according to FIGO (*p* < 621) (Table 7).

In the following, the study group was limited to patients with ovarian cancer (III–IV stage according to FIGO), which is clinically significant due to the risk of relapse.

There was an evaluation of endorphin and serotonin levels before and after chemotherapy depending on the occurrence of relapse. A Student’s *t*-test was performed for independent samples. A statistically significant difference was demonstrated in the levels of β-endorphins before (*p* < 0.016) and after (*p* < 0.039) chemotherapy in the study group. A lower level of β-endorphins was found in the patients with a recurrence of the neoplastic process. On the other hand, the level of serotonin before chemotherapy was higher in the group of people who had relapsed. There was no statistically significant difference in the levels of serotonin before (*p* < 0.066) and after chemotherapy (*p* < 0.482) (Table 8).

The relationship was also assessed between β-endorphin and serotonin levels and disease-free time (PFS—progression-free survival) in the study group with ovarian cancer (III–IV stage according to FIGO) The analysis of the correlation of the Spearman’s rank coefficient, taking into account the levels of both substances in the measurement before and after chemotherapy, showed a statistically significant positive correlation between the level of β-endorphin and disease-free time (β-endorphin levels before chemotherapy: rho Spearman 0.421, *p* < 0.044; β-endorphin levels after chemotherapy: rho Spearman 0.523 *p* < 0.009). This means that the higher the β-endorphin level (both before and after chemotherapy), the longer the disease-free time could be seen. On the other hand, the level of serotonin after chemotherapy positively correlated with PFS (serotonin levels before chemotherapy: rho Spearman 0.366, *p* < 0.041). Pre-chemotherapy serotonin levels were not significantly associated with PFS (serotonin levels before chemotherapy: rho Spearman-0.093, *p* < 0.497)—Table 9.

Then, the levels of β-endorphin and serotonin were assessed depending on the overall survival of the patients. A Student’s *t*-test was performed for independent samples, taking into account the level of both substances before and after chemotherapy. Endorphin levels before (*p* < 0.022) and after (*p* < 0.008) chemotherapy were higher in the group of survivors. There were no statistically significant differences in serotonin levels—before (*p* < 0.414) and after chemotherapy (*p* < 0.818)—Table 10.

The relationship between β-endorphin and serotonin levels and survival time was investigated. The analysis of the Spearman’s correlation rank coefficient, taking into account the level of both substances in the measurement before and after chemotherapy, showed a statistically significant positive correlation between the level of β-endorphin and survival time (β-endorphin levels before chemotherapy: rho Spearman 0.184, *p* < 0.031; β-endorphin levels after chemotherapy: rho Spearman 0.507, *p* < 0.011). This means that the higher the level of β-endorphin (both before and after chemotherapy), the longer the respondents’ life could be seen. Serotonin levels were not significantly correlated with survival time (serotonin levels before chemotherapy: rho Spearman -0.129, *p* < 0.296; serotonin levels after chemotherapy: rho Spearman 0.152, *p* < 0.533)—Table 11.

Univariate analysis showed a statistically significant correlation between disease-free time and overall survival in the study group, the stage of ovarian cancer, and the level of endorphin. Additionally, there was a statistically significant correlation between overall survival and age, as well as serotonin above the median, serotonin level above 95%, and pain level. There was also a statistically significant correlation between disease-free time and the beta-endorphin level above 95%. There was no statistically significant correlation between disease-free time and overall survival in the study group and, other variables (grade 1 vs. 3; beta-endorphins 75%; serotonin 75%). Multivariate analysis showed a statistically significant relationship between the disease-free time and overall survival in the patients, age, the stage of ovarian cancer, an endorphin level above the median and a serotonin level above 95%. There was also a statistically significant correlation between disease-free time and serotonin levels above 95%. There was no statistically significant relationship between disease-free time and overall survival in the study group and other variables (grade 1 vs. 3; β-endorphins 75%; serotonin level above median; serotonin level above 75%) (Table 12).

## 4. Discussion

Ovarian cancer is a type of gynecological neoplasm with the highest mortality. Information about a positive cancer diagnosis is the cause of many negative emotions in patients that relate to anxiety, social isolation, psychological stress, and depression. These factors not only worsen the effectiveness of the therapy but also contribute to faster development of the neoplasm [20,21]. The stress that appears following a positive cancer diagnosis leads to disorders of the control mechanism, impairing the immune system and often leading to disease progression. Research indicates that the tumor growth rate and incidence are associated with chronic stress in patients [22].

Our research shown that patients with ovarian cancer had much lower levels of endorphin than the patients from the control group. It was also shown that the level of β-endorphins both before and after chemotherapy was higher in the group of survivors.

Similarly, Shrihari’s research points out that, in patients who lead a lifestyle conducive to the production of β-endorphins, the risk of developing ovarian cancer is reduced. Such activities include physical activity, meditation, music therapy and acupuncture [13].

Sakar et al. in a study on rats, showed the role of β-endorphin in the inhibition of tumor progression, presumably due to a reduction in the production of catecholamines and inflammatory cytokines such as IL-1α, IL-12, and tumor necrosis factor α (TNF-α). This may be related to the inhibition of sympathetic neuronal function by β-endorphins, resulting in an increase in the activity of peripheral NK cells and macrophages [3,23]. The latest reports in the literature suggest the anti-tumor activity of β-endorphins due to the activation of interferon gamma (IFN-gamma), granzyme-B, and perforin mediated by NK cells and macrophages. They activate the antiviral potential by changing the apoptotic activity and the decrease in cellular proliferation. Thanks to this, the gene expression environment in the tumor microenvironment becomes unfavorable for the development of the carcinogenesis process [3,12,13].

This study on serotonin concentration, both in the research and control groups, shows similar results. It is worth emphasizing that in both groups the levels assumed as the standard concentration were recorded. Additionally, the level of serotonin was not significantly related to the patient’s survival.

A study by Qin et al. demonstrated the important role of serotonin/HTR1E signaling in the prevention of ovarian progression cancer (OC) caused by chronic psychological stress, suggesting the potential therapeutic value of a specific HTR1E agonist and SRC inhibitor for patients with OC (ovarian cancer) suffering from mental stress [21]. Henriksen et al. found that serotonin (5-HT) receptors 5-HTR1A, 5-HTR1B, 5-HTR2B, and 5-HTR4 are expressed in the healthy ovary as well as in malignant tumors of the ovary. These receptors have been shown to be overexpressed in mild and non-invasive tumors, while their expression is downgraded in more invasive forms of ovarian tumors [24]. Other studies have shown that various ovarian cancer cell lines (SKOV3, HEYA8, 2774, ES2, TOV112D, OV90, SW626, UWB1.298, and CaOV3) also overexpress serotonin receptors 5-HTR1A, 5-HTR1B, 5-HTR1D and 5-HTR2A. However, only the SW626, UWB1.298, and CaOV3 cell lines overexpress 5-HTR2B compared to normal ovarian cells. Christensen et al. showed that treatment with both serotonin and a 5-HTR2A agonist increased proliferation and survival of the ovarian cancer cell lines SKOV3, CP20, and ES2. They also showed that injection of serotonin and the SSRI, sertraline, increased tumor mass and Ki67 expression in a SKOV3 tumor model in athymic nude mice [25]. These findings suggest that serotonin signaling promotes the development of ovarian cancer. Until recently, serotonin was thought to be mainly limited to regulating body functions through concomitant neurons in the CNS and the peripheral nervous system. It turns out that other cells, such as immune cells and cancer cells, also synthesize, release, and respond to serotonin. Despite reports of the conflicting role of serotonin in regulating the function of immune cells, most studies confirm that serotonin plays a role in enhancing their anti-inflammatory function through increased secretion of anti-inflammatory cytokines. This suggests that serotonin may have a pro-tumor effect [15].

This present study also analyzed the levels of β-endorphin, serotonin, and the incidence of recurrence in patients. It was shown that the serotonin level was higher in patients who relapsed before chemotherapy than in the patients from the control group. Patients with a relapse of the neoplastic process had lower levels of β-endorphins. This applied both to patients treated with chemotherapy and those not.

These results may support the hypothesis concerning the protective function of β-endorphins in the pathogenesis of ovarian cancer. The state of the disease is undoubtedly a source of high stress compounded throughout the treatment period and numerous side effects. Studies carried out on animal and human models indicate that events causing severe stress have a negative impact on the functioning of the immune system in the form of disturbances in the transport mechanisms of neutrophils, macrophages, T and B lymphocytes [26]. In addition, Morvan and Lanier’s research has shown that stress and depression may be responsible for the first line of carcinogenesis processes due to the reduced activity of NK cells. It has been shown that these cells constitute the first protective barrier preventing metastatic processes, and their low activity leads to the growth of neoplasms in both humans and animals [27]. Moreover, Shrihari showed that β-endorphins inhibit the activation of NF-ĸB, which is a key transcription factor responsible for tumor progression [28]. The above studies are particularly important in the context of chronic stress experienced by patients in connection with a history of cancer. A meta-analysis by Moreno-Smith et al. indicates the role of stress in the pathogenesis of various types of cancer. Long exposure to a stress factor activates specific signaling pathways both in neoplastic cells and in the tumor microenvironment, leading to its growth and progression [29]. That is why it seems so important not only to care for patients during treatment but also after its completion, when patients may still experience negative psychological effects in the form of fear of recurrence, depression related to complications, and hormonal fluctuations.

This study had some limitations. A significant limitation of this study was the small group of respondents, and further studies with an appropriate sample size are needed to verify the presented data. Despite the fact, that not all patients were at the same stage of clinical advancement of the tumor and the degree of histopathological differentiation of the tumor. This is the majority of patients, because as many as 68 were qualified for the high-grade group and advanced in terms of staging, which gives an almost homogeneous group of patients in terms of psychology. All patients with ovarian cancer after surgery received standard chemotherapy based on platinum and paclitaxel. On the other hand, patients with benign lesions in the ovary were only treated with surgery.

## 5. Conclusions

This study shows that the levels of serotonin and β-endorphin levels are significantly related to the ovarian cancer and change during treatment. The level of β-endorphins and serotonin are factors that can influence disease-free survival as well as overall survival. In patients with shorter survival times, lower levels of β-endorphins were noted. This applied to patients who were undergoing chemotherapy as well as patients which had a relapse. A comparison of endorphin and serotonin levels between patients and controls showed low levels of β-endorphin in patients with ovarian cancer. Patients with a low concentration of β-endorphins may have a weakened immune system susceptible to carcinogenesis processes. This study suggests that modifiable factors such as β-endorphins and serotonin levels may affect the course of treatment, disease-free time, and overall survival.

## Figures and Tables

**Table 1:** Basic descriptive statistics of the researched quantitative variables.

	M	Me	SD	Sk.	Kurt.	Min	Max	D	*p*
β-endorphin measurement I	246.37	220.90	53.27	0.34	−1.51	158.90	354.20	0.21	<0.001
β-endorphin measurement II	194.31	195.89	18.71	−0.07	−0.26	143.83	231.71	0.06	0.200
Serotonin measurement I	141	136.80	18.15	1.06	2.81	90.10	213.40	0.13	<0.001
Serotonin measurement II	135.24	136.70	20.36	0.19	0.01	95.40	183.40	0.09	0.167
Months to relapse	16.76	17.50	5	−0.10	2.49	3	30	0.14	0.083
Survival months	32.64	36	5.48	−1.88	3.34	10	36	0.31	<0.001

M—median; Me—median; SD—standard deviation; Sk.—skewness; Kurt.—kurtosis; Min and Max—lowest and highest value of the distribution; D—the result of the Kolmogorov-Smirnov test; *p*—significance.

**Table 2:** The preoperative levels of β-endorphin in the study group (with ovarian cancer—I–II stage according to FIGO) and the control group (with benign neoplastic lesions and simple ovarian cysts).

	FIGO I,II (*n* = 10)		Control Group (*n* = 61)				95% CI		
	M	SD	M	SD	Vol	*p*	LL	UL	d Cohen
β-endorphin levels	223.6	16.08	302.00	24.49	−31.26	0.003	−105.41	−93.49	5.03

M—median; SD—standard deviation; *p*—significance level; 95% CI—confidence interval, LL—lower limit; UL—upper limit; d Cohen—the size of the effect.

**Table 3:** The preoperative levels of β-endorphin in the study group (with ovarian cancer—III–IV stage according to FIGO) and the control group (with benign neoplastic lesions and simple ovarian cysts).

	FIGO III,IV (*n* = 68)		Control Group (*n* = 61)				95% CI		
	M	SD	M	SD	Vol	*p*	LL	UL	d Cohen
β-endorphin levels	208.6	14.23	302.00	24.49	−28.67	<0.001	−102.39	−91.98	4.96

M—median; SD—standard deviation; *p*—significance level; 95% CI—confidence interval, LL—lower limit; UL—upper limit; d Cohen—the size of the effect.

**Table 4:** The preoperative levels of β-endorphin in the group with ovarian cancer—III–IV stage according to FIGO and the group with ovarian cancer—I–II stage according to FIGO.

	FIGO III,IV (*n* = 68)		FIGO I,II (*n* = 10)				95% CI		
	M	SD	M	SD	Vol	*p*	LL	UL	d Cohen
β-endorphin levels	208.6	14.23	223.6	16.08	−25.69	0.342	−91.24	−87.62	3.99

M—median; SD—standard deviation; *p*—significance level; 95% CI—confidence interval, LL—lower limit; UL—upper limit; d Cohen—the size of the effect.

**Table 5:** Serotonin preoperative levels in the study group (with ovarian cancer—I–II stage according to FIGO) and the control group (with benign neoplastic lesions and simple ovarian cysts).

	FIGO I,II (*n* = 10)		Control Group (*n* = 61)				95% CI		
	M	SD	M	SD	Vol	*p*	LL	UL	d Cohen
Serotonin levels	134.62	10.93	145.15	22.66	−2.39	0.038	−12.96	−0.89	0.56

M—median; SD—standard deviation; *p*—significance level; 95% CI—confidence interval, LL—lower limit; UL—upper limit; d Cohen—the size of the effect.

**Table 6:** Serotonin preoperative levels in the study group (with ovarian cancer—III–IV stage according to FIGO) and the control group (with benign neoplastic lesions and simple ovarian cysts).

	FIGO III,IV (*n* = 68)		Control Group (*n* = 61)				95% CI		
	M	SD	M	SD	vol	*p*	LL	UL	d Cohen
Serotonin levels	136.22	11.96	145.15	22.66	−2.31	0.048	−13.99	−1.03	0.48

M—median; SD—standard deviation; *p*—significance level; 95% CI—confidence interval, LL—lower limit; UL—upper limit; d Cohen—the size of the effect.

**Table 7:** The preoperative levels of serotonin in the group with ovarian cancer—III–IV stage according to FIGO and the group with ovarian cancer—I–II stage according to FIGO.

	FIGO III,IV (*n* = 68)		FIGO I,II (*n* = 10)				95% CI		
	M	SD	M	SD	Vol	*p*	LL	UL	d Cohen
Serotonin levels	136.22	11.96	134.62	10.93	−2.01	0.621	−11.87	−0.66	0.21

M—median; SD—standard deviation; *p*—significance level; 95% CI—confidence interval, LL—lower limit; UL—upper limit; d Cohen—the size of the effect.

**Table 8:** The level of β-endorphin and serotonin and the recurrence in the study group with ovarian cancer (III–IV stage according to FIGO).

	No (*n* = 36)		Yes (*n* = 32)				95% CI		
	M	SD	M	SD	Vol	*p*	LL	UL	d Cohen
β-endorphin levels before chemotherapy	219.62	19.26	193.21	13.99	3.96	0.016	6.5	20.02	0.91
β-endorphin level after chemotherapy	209.54	15.01	179.02	13.01	7.99	0.039	20.06	31.05	2.30
Serotonin levels before chemotherapy	136.21	11.11	140.86	14.67	−3.02	0.066	−10.66	−0.42	0.60
Serotonin levels after chemotherapy	129.87	18.23	138.12	20.89	−1.09	0.482	−12.89	7.23	0.22

M—median; SD—standard deviation; *p*—significance level; 95% CI—confidence interval, LL—lower limit; UL—upper limit; d Cohen—the size of the effect.

**Table 9:** Relationship of β-endorphin, serotonin, and PFS levels in the group- with ovarian cancer (III–IV stage according to FIGO).

		Months to Relapse
β-endorphin levels before chemotherapy	rho Spearman	0.421
	*p*	0.044
Serotonin levels before chemotherapy	rho Spearman	−0.093
	*p*	0.497
β-endorphin levels after chemotherapy	rho Spearman	0.523
	*p*	0.009
Serotonin levels after chemotherapy	rho Spearman	0.366
	*p*	0.041

*p*—significance level; rho Spearman—Spearman’s rank correlation coefficient.

**Table 10:** The level of β-endorphins, serotonin, and the survival of the women in the study group- with ovarian cancer (III–IV stage according to FIGO).

	No (*n* = 32)		Yes (*n* = 36)				95% CI		
	M	SD	M	SD	Vol	*p*	LL	UL	d Cohen
β-endorphin levels before chemotherapy	193.22	13.70	220.34	15.02	−4.16	0.022	−20.02	−7.54	0.88
β-endorphin levels after chemotherapy	177.12	12.02	210.29	14.34	−8.12	0.008	−31.07	−19.02	1.55
Serotonin levels before chemotherapy	139.23	13.79	135.44	13.01	0.55	0.414	−4.86	7.51	0.21
Serotonin levels after chemotherapy	136.11	23.02	135.21	19.22	0.34	0.818	−7.56	10.26	0.19

M—median; SD—standard deviation; *p*—significance level; 95% CI—confidence interval, LL—lower limit; UL—upper limit; d Cohen—the size of the effect.

**Table 11:** Relationship of β-endorphin and serotonin levels and survival time in the study group- with ovarian cancer (III–IV stage according to FIGO).

		Survival Time
β-endorphin levels before chemotherapy	rho Spearman	0.184
	*p*	0.031
Serotonin levels before chemotherapy	rho Spearman	−0.129
	*p*	0.296
β-endorphin levels after chemotherapy	rho Spearman	0.507
	*p*	0.011
Serotonin levels after chemotherapy	rho Spearman	0.152
	*p*	0.533

*p*—significance level; rho Spearman—Spearman’s rank correlation coefficient.

**Table 12:** Survival analysis of the study group- women with ovarian cancer (III–IV stage according to FIGO).

	**Univariate Analysis**					
	**PFS**			**OS**		
	**HR**	**95% CI**	***p*-Value**	**HR**	**95% CI**	***p*-Value**
Age	1.14	1.08–1.20	0.059	1.13	1.11–1.20	0.039
FIGO	1.37	1.30–1.39	0.016	1.30	1.29–1.34	0.020
Grade 1 vs. 3	1.40	1.28–1.40	0.486	1.27	1.25–1.31	0.076
Pain level	1.09	1.01–1.14	0.088	1.08	1.06–1.09	0.043
beta-endorphins median	0.81	0.75–0.88	0.039	0.90	0.83–0.93	0.041
Serotonin median	0.90	0.88–1.05	0.202	1.05	0.92–1.10	0.048
beta-endorphins 75%	0.92	0.82–0.93	0.289	0.82	0.81–0.92	0.205
Serotonin 75%	1.01	0.83–1.04	0.567	1.15	1.09–1.24	0.327
beta-endorphin 95%	0.87	0.86–0.95	0.021	0.84	0.79–0.92	0.064
Serotonin 95%	0.96	0.82–0.99	0.631	1.02	1.01–1.10	0.037
	**Multivariate Analysis**					
	**PFS**			**OS**		
	**HR**	**95% CI**	***p*-Value**	**HR**	**95% CI**	***p*-Value**
Age	1.11	1.04–1.19	0.014	1.20	1.11–1.23	0.005
FIGO	1.31	1.23–1.33	0.004	1.24	1.17–1.26	0.008
Grade 1 vs. 3	1.05	1.01–1.13	0.502	1.02	0.98–1.06	0.523
β-endorphins	0.82	0.74–0.85	0.049	0.88	0.82–0.90	0.009
β-endorphins 75%	0.93	0.87–1.08	0.212	1.08	1.02–1.10	0.082
β-endorphin 95%	0.83	0.82–0.86	0.046	0.91	0.88–1.01	0.073
Serotonin median	0.93	0.93–1.10	0.418	0.92	0.89–0.98	0.189
Serotonin 75%	1.12	1.07–1.20	0.282	1.00	0.95–1.05	0.326
Serotonin 95%	1.06	0.99–1.12	0.020	1.09	1.01–1.11	0.006

PFS—progression-free survival—disease-free time; OS—overall survival—total survival time; HR—hazard ratio; 95% CI—confidence interval; *p*-value—test similarity.

## Data Availability

The data presented in this study are available on request from the last author.

## Appendix A

**Table A1:** Basic descriptive statistics of the researched quantitative variables.

	**M**	**Me**	**SD**	**Sk.**	**Kurt.**	**Min**	**Max**	**D**	***p***
**Coping Inventory For Stressful Situations (CISS)**									
SSZ-Task-Oriented Style	50.86	50	8.45	0.04	−0.27	32	69	0.08	0.200
SSE-Emotion-Oriented Style	44.13	45	9.46	−0.49	−0.31	21	64	0.13	0.003
SSU-Avoidant Style	47.38	48	6.86	−0.44	0.28	29	63	0.10	0.070
ACZ-Distraction Seeking	22.52	22	5.54	−0.11	−0.52	11	36	0.08	0.200
PKT-Social Diversion.	16.08	16	3.10	0.43	0.17	9	24	0.10	0.032
**Satisfaction with Life Scale (SWLS)**									
Satisfaction With Life	23.25	23	3.86	0.02	−0.47	15	33	0.08	0.200
**Beck’s Depression Inventory (BDII)**									
Depression Level	24.19	29	12	−0.44	−1.05	0	46	0.16	<0.001
**Multidimensional Health Locus of Control A (MHLC A)**									
Internal (A)	21.39	22	4.37	−0.13	−0.15	11	33	0.09	0.200
Chance (A)	22.48	22	5.04	−0.19	−0.36	10	34	0.10	0.051
Powerful others (A)	20.66	21	4.80	−0.14	0.06	6	31	0.08	0.200
**Multidimensional Health Locus of Control B (MHLC B)**									
Internal (A)	21.62	21	4.97	0.22	−0.08	11	36	0.07	0.200
Chance (A)	22.87	22	5.33	0.16	−0.18	10	36	0.10	0.065
Powerful others (A)	22.01	22	4.09	0.15	0.26	11	33	0.10	0.045
**General Self-Efficacy Scale (GSES)**									
Self-efficacy	27.22	27	4.28	−0.21	0.31	15	38	0.10	0.048
**Stress Sensation Questionnaire (KPS)**									
Emotional tension	21.33	22	5.03	−0.79	0.45	7	30	0.12	0.010
External stress,	19.99	20	4.65	−0.77	0.63	7	28	0.11	0.025
Intrapsychic stress	20.91	21	4.51	−0.68	0.09	8	28	0.13	0.004
Generalized stress level	62.23	64	10.57	−1.26	2.49	24	78	0.12	0.008

M—median; Me—median; SD—standard deviation; Sk.—skewness; Kurt.—kurtosis; Min and Max—lowest and highest value of the distribution; D—the result of the Kolmogorov-Smirnov test; *p*—significance.

## References

[1] Webb P.M., Jordan S.J. (2017). Epidemiology of epithelial ovarian cancer. Best Pract. Res. Clin. Obstet. Gynaecol..

[2] Kujawa K.A., Lisowska K.M. (2015). Rak jajnika–od biologii do kliniki. Postepy Hig. Med. Dosw..

[3] Asvadi N.H., Morgan M., Hewavitharana A.K., Shaw P.N., Cabot P.J. (2014). Biotransformation of beta-endorphin and possible therapeutic implications. Front. Pharmacol..

[4] Saba G.C. (2011). The immune-endocrine system: Hormones, receptors and endocrine function of immune cells-The packed transport theory. Adv. Neuroimmune Biol..

[5] Fancourt D., Ockelford A., Belai A. (2014). The psychoneuroimmunological effects of music: A systematic review and a new model. Brain Behav. Immun..

[6] Shrihari T.G. (2017). Endorphins on cancer: A novel therapeutic approach. J. Carcinog. Mutagen..

[7] Kiecolt-Glaser J.K., Bennet J.M., Andridge R., Peng J., Shapiro C.L., Malarkey W.B., Emery C.F., Layman R., Mrozek E.E., Glaser R. (2014). Yoga’s impact on inflammation, mood, and fatigue in breast cancer survivors; A randomized controlled trial. J. Clin. Oncol..

[8] Nani M., Irwin M.R., Chung M., Wang C. (2014). The effect of mind–body therapies on the immune system–meta analysis. PLoS ONE.

[9] Priyadarshini S., Palok A. (2012). Effects of psychological stress on innate immunity and metabolism in humans: A systematic analysis. PLoS ONE.

[10] Hua S. (2016). Neuroimmune interaction in the regulation of peripheral opioid mediated analgesia in inflammation. Front. Immunol..

[11] Sedlmeir P., Eberth J., Schwar Z.M., Zimmermann D., Haarig F., Jaeger S., Kunze S. (2012). The psychological effects of meditations: A meta–Analysis. Psychol. Bull..

[12] Sarkar D.K., Murugan S., Zhang C., Boyadjieva N. (2012). Regulation of cancer progression by β-endorphin neuron. Cancer Res..

[13] Shrihari T.G. (2018). Endorphins- A novel hidden magic holistic healer. J. Clin. Cell Immunol..

[14] Iwaszkiewicz K.S. (2013). Targeting peripheral opioid receptors to promote an analgesic and anti-inflammatory action. Front. Pharmacol..

[15] Karmakar S., Lal G. (2021). Role of serotonin receptor signaling in cancer cells and anti-tumor immunity. Theranostics.

[16] Berger M., Gray J.A., Roth B.L. (2009). The Expanded Biology of Serotonin. Annu. Rev. Med..

[17] Sarrouilhe D., Clarhaut J., Defamie N., Mesnil M. (2015). Serotonin and cancer: What is the link?. Curr. Mol. Med..

[18] (2021). FIGO Cancer Report. https://www.figo.org/figo-cancer-report-2021.

[19] George D., Mallery P. (2019). IBM SPSS Statistics 26 Step by Step: A Simple Guide and Reference.

[20] Faller H., Schuler M., Richard M., Heckl U., Weis J., Küffner R. (2013). Effects of Psycho-Oncologic Interventions on Emotional Distress and Quality of Life in Adult Patients With Cancer: Systematic Review and Meta-Analysis. J. Clin. Oncol..

[21] Qin X., Li J., Wang S., Lv J., Luan F., Liu Y., Chen Y., Chen X., Zhao Y., Zhu J. (2021). Serotonin/HTR1E signaling blocks chronic stress-promoted progression of ovarian cancer. Theranostics.

[22] Zhang C., Murugan S., Boyadjieva N., Jabbar S., Shrivastava P., Sarkar D.K. (2014). Beta-Endorphin Cell Therapy for Cancer Prevention. Cancer Prev. Res..

[23] Sarkar D.K., Zhang C., Murugan S., Dokur M., Boyadjieva N.I., Ortiguela M., Kenneth R., Mojtehedzadeh S. (2011). Transplantation of Endorphin Neurons into the Hypothalamus Promotes Immune Function and Restricts the Growth and Metastasis of Mammary Carcinoma. Cancer Res..

[24] Henriksen R., Dizeyi N., Abrahamsson P.A. (2012). Expression of serotonin receptors 5-HT1A, 5-HT1B, 5-HT2B and 5-HT4 in ovary and in ovarian tumors. Anticancer Res..

[25] Christensen D.K., Armaiz-Pena G.N., Ramirez E., Matsuo K., Zimmerman B., Zand B., Shinn E., Goodheart M.J., Bender D., Thaker P.H. (2016). SSRI use and clinical outcomes in epithelial ovarian cancer. Oncotarget.

[26] Capuron L., Miller A.H. (2011). Immune system to brain signaling: Neuropsychopharmacological implications. Clin. Pharmacol. Ther..

[27] Morvan M.G., Lanier L.L. (2016). NK cells and cancer: You can teach innate cells new tricks. Nat. Rev. Cancer.

[28] Shrihari T.G. (2019). Beta endorphins-holistic therapeutic approach to cancer. Ann. Ib. Postgrad. Med..

[29] Moreno-Smith M., Lutgendorf S.K., Sood A.K. (2010). Impact of stress on cancer metastasis. Future Oncol..

